# Galectin-3: A Link between Myocardial and Arterial Stiffening in Patients with Acute Decompensated Heart Failure?

**DOI:** 10.5935/abc.20150149

**Published:** 2016-02

**Authors:** Radu Ioan Lala, Dan Darabantiu, Luminita Pilat, Maria Puschita

**Affiliations:** 1 "Vasile Goldis" West University Arad – Romania; 2 Arad County Emergency Clinical Hospital – Cardiology Department – Romania

**Keywords:** Galectin 3/analysis, Biomarkers Pharmacological, Vascular Stiffness, Heart Failure/pathology, Heart Ventricles/physiopathology, Echocardiography

## Abstract

**Background:**

Heart failure is accompanied by abnormalities in ventricular-vascular interaction due to increased myocardial and arterial stiffness. Galectin-3 is a recently discovered biomarker that plays an important role in myocardial and vascular fibrosis and heart failure progression.

**Objectives:**

The aim of this study was to determine whether galectin-3 is correlated with arterial stiffening markers and impaired ventricular-arterial coupling in decompensated heart failure patients.

**Methods:**

A total of 79 inpatients with acute decompensated heart failure were evaluated. Serum galectin-3 was determined at baseline, and during admission, transthoracic echocardiography and measurements of vascular indices by Doppler ultrasonography were performed.

**Results:**

Elevated pulse wave velocity and low arterial carotid distensibility are associated with heart failure in patients with preserved ejection fraction (p = 0.04, p = 0.009). Pulse wave velocity, carotid distensibility and Young’s modulus did not correlate with serum galectin-3 levels. Conversely, raised galectin-3 levels correlated with an increased ventricular-arterial coupling ratio (Ea/Elv) p = 0.047, OR = 1.9, 95% CI (1.0‑3.6). Increased galectin-3 levels were associated with lower rates of left ventricular pressure rise in early systole (dp/dt) (p=0.018) and raised pulmonary artery pressure (p = 0.046). High galectin-3 levels (p = 0.038, HR = 3.07) and arterial pulmonary pressure (p = 0.007, HR = 1.06) were found to be independent risk factors for all-cause mortality and readmissions.

**Conclusions:**

This study showed no significant correlation between serum galectin-3 levels and arterial stiffening markers. Instead, high galectin-3 levels predicted impaired ventricular-arterial coupling. Galectin-3 may be predictive of raised pulmonary artery pressures. Elevated galectin-3 levels correlate with severe systolic dysfunction and together with pulmonary hypertension are independent markers of outcome.

## Introduction

Heart failure is a complex syndrome characterized by continuous molecular, cellular and interstitial changes that lead to changes in size, shape and function of the heart. Part of these changes are due to exaggerated neurohormonal activation which has been found to have a direct relationship with arterial stiffness.^1^ Several studies have shown that arterial stiffness is increased in congestive heart failure, and is associated with left ventricle (LV) diastolic dysfunction.^2,3^ These findings are particularly important because reduced arterial compliance is responsible for abnormal ventricular-arterial coupling, greater cardiac afterload, increased LV wall stress and reduced coronary flow, all of which lead to deterioration in LV function.^4^

Recent attention has been focused on one emerging biomarker, galectin-3, which is distributed in various cells including macrophages and is up-regulated during inflammation.^5^ Studies have pointed out a close relationship between macrophage activation and fibrosis in heart failure pathogenesis.^6^ Galectin-3 has been proved to be involved in fibrosis, hypertrophy and heart failure progression.^7^ Given the aforementioned pathogenic mechanisms and the pro-inflammatory state that is found in heart failure, we considered galectin-3 a possible mediator of vascular fibrosis and a link between myocardial and arterial stiffening. Therefore we aim to evaluate whether galectin-3 correlates with arterial stiffening markers and impaired ventricular-arterial coupling in decompensated heart failure patients.

## Methods

This study was conducted in 2014. Seventy-nine inpatients were evaluated at the clinical county hospital with a primary diagnosis of acute decompensated heart failure. Serum galectin-3 of patients was determined at baseline. Of this cohort, 60 patients had heart failure with reduced ejection fraction (EF) (mean of 25%) and 19 patients had heart failure with preserved EF (mean of 45%). The study was approved by the hospital Medical Ethics Committee and complied with the Helsinki Declaration. Written informed consent was obtained from all patients.

### Echocardiography (ECG)

Transthoracic ECG was performed on each patient during hospitalization. The following structural indices were assessed: left ventricular (LV) end-diastolic and systolic volumes and diameters, LV wall thickness, LV mass (Devereux equation), relative wall thickness (RWT), left atrial indexed volume, pulmonary artery systolic pressure (PASP). Markers of systolic function included determination of LV EF by the biplane modified Simpson's method, fractional shortening and the rate of LV pressure rise in early systole (dp/dt). Markers of diastolic dysfunction were also determined: transmitral diastolic velocities (E, A), transmitral flow propagation velocity (Vp), early deceleration time (DTE), septal mitral annulus velocities (e', a') by tissue Doppler, diastolic ratios E/A, E/e', and E/Vp. Each patient was evaluated using the same echo-machine, and two investigators analyzed the echocardiographic data.

### Vascular Function Indices

The following vascular indices were assessed: pulse wave velocity, carotid distensibility coefficient and Young's elastic modulus. Each of these is considered to provide reliable parameters when evaluating arterial stiffness. Pulse wave velocity was measured by pulsed wave Doppler ultrasound probe synchronized with the ECG. Common carotid artery was located by placing the sample volume at the supraclavicular level, and the common femoral artery by placing the sample volume in the groin (not simultaneously). After acquisition of Doppler tracing, we made three consecutive recordings from both arteries, and measured the time from the R wave of the QRS to the foot of the Doppler waveform using digital calipers (the time taken by the blood ejected from the heart to reach the studied arteries). We calculated the average time of the three recordings, and measured the distance between the sites where we traced the Doppler waveforms. For the carotid distensibility coefficient and Young's modulus, we used the M-mode echo to measure the diameter of the vessel during systole and diastole, and the vessel's wall thickness. Finally, we measured the intima medial thickness by echo-2D. These parameters were calculated by existing mathematical formulas: Distensibility coefficient = (Dmax^2^-Dmin^2^)/Dmax*MAP; Young Modulus = MAP*Dmax/ (Dmax-Dmin)*h; Pulse wave velocity = Dist/T_2_ -T_1_, where Dmax: maximum diameter of the vessel during systole, Dmin: minimum diameter of the vessel during diastole, MAP: mean arterial pressure, h: thickness of the vessel wall, Dist: distance between arterial carotid site and arterial femoral site, T_2_: average time between R wave of the QRS and the beginning of the Doppler waveform of the common femoral artery, T_1_: average time between R wave of the QRS and the beginning of the Doppler waveform of the common carotid artery.

### Arterial-ventricular coupling

Brachial blood pressure was determined with patient at rest, using a sphygmomanometer and a standard stethoscope. Arterial elastance (Ea) is a parameter used to estimate the arterial load which represents the change in volume for any change in pressure. The Ea index includes characteristics such as arterial compliance and peripheral vascular resistance, and is calculated as Ea = end-systolic pressure/stroke volume. A non-invasive estimation of the end-systolic pressure was made by using the approximation formula: 0.9 x brachial systolic pressure, which accurately predicts pressure-volume loop measurements of end-systolic pressure.^8^ Stroke volume was determined echocardiographically by Simpson's modified biplane method. Next we evaluated the LV elastance (Elv) index which is a parameter used to estimate the LV contractility based on the following equation Elv = end-systolic pressure/ end-systolic volume - V_0_, where V_0_ is the theoretical volume at zero pressure, and considered to be negligible compared to end-systolic volume.^8^ Finally we evaluated arterial-ventricular coupling by determining the Ea/Elv ratio, which estimates cardiovascular hemodynamics through the interaction between the arterial system and the left ventricle.

### Laboratory assessment

Blood samples were collected during admission. The samples were centrifuged, and serum galectin-3 concentration was determined using an optimized enzyme-linked immunosorbent assay kit (Human galectin-3 NBP1-91275, NOVUS BIOLOGICALS, R&D Systems Europe, Germany), and measured using a Tecan Sunrise microplate reader. This is an immunoassay in which a biotin-conjugated anti-human galectin-3 antibody binds to human galectin-3 captured by the coating antibody. Calibration and standardization of the assay were performed according to the manufacturer's protocol.

### Statistical analyses

Continuous variables with normal distribution are presented as means and standard deviation, and significance of differences was tested by Student's t-test. Continuous variables with non-normal distribution are expressed as medians (interquartile range, IQR), and significance of differences was tested by Kruskal-Wallis test or the Mann-Whitney U test. Categorical variables are expressed as percentages. Normality of distribution was evaluated by Shapiro-Wilk test. Correlations of vascular indices with clinical and echocardiographic variables were assessed using the Spearman correlation analysis. We also used Pearson's chi-square test for comparison of categorical values, multivariate logistic regression to assess the relationship between dependent categorical variables and independent variables, and Cox regression survival analysis to identifying predictors of composite endpoint (all-cause mortality and readmissions). All the analyses were performed using the IBM SPSS 20 package. Statistical significance was assessed by two-tailed test and a p < 0.05 was considered significant.

## Results

Mean age of the cohort was 64 ± 11 years of which 73% were males and 26% were females. During a 12-month follow-up, 35 readmissions for worsening of heart failure and 1 death (cardiogenic shock) were recorded. First, we divided the patients in two groups on the basis EF, reduced or preserved (< 40% or ≥ 40%, respectively) (Table 1). Median serum levels of galectin-3 were not significantly different (p = 0.9) between patients with heart failure with reduced EF (8.2 ng/ml [3.6-14.0]) (median and IQR) and those with heart failure with preserved EF (9.7 ng/ml [3.7-12.0]). Compared with patients with reduced EF, patients with preserved EF presented significantly higher pulse wave velocities (11.3 m/s [9.4-16.0] versus 10 m/s [7.3-12.5], p = 0.04), systolic blood pressure (169 ± 45 mmHg versus 134 ± 60 mmHg, p = 0.004), and age (76 years versus 62 years, p = 0.001), and lower carotid distensibility coefficient (2.7 mmHg [1.8-3.5] versus 3.9 mmHg [2.7-5.7], p = 0.009) (Table 2).

**Table 1:** Heart failure etiology and associated conditions

**Heart failure etiology**	**Ejection fraction**		
	**HFPEF**	**HFREF**	**p**
Ischemic n(%)	9(47.4)	27(45)	0.85
Hypertension n(%)	8(42.1)	12(20)	0.05
Valvular n(%)	2(10.5)	2(3.3)	0.21
Idiopathic dilated cardiomyopathy n(%)	0(0)	19(31.7)	0.005
**Decompensated heart failure**			
Ischemic event n(%)	4(21.1)	12(20)	0.92
Hypertensive pulmonary edema n(%)	11(57.9)	2(3.3)	< 0.001
Arrhythmia n(%)	3(15.8)	5(8.3)	0.34
Valvular disease n(%)	1(5.3)	4(6.7)	0.82
Poor medication adherence n(%)	0(0)	10(16.7)	0.05
Increased sodium intake n(%)	0(0)	11(18.3)	0.04
Other n(%)	0(0)	15(25)	0.01
Readmissions n(%)	13(47.4)	22(26.6)	0.05
Diabetes n(%)	11(57.9)	24(40)	0.1
Atrial fibrillation n(%)	8(42.1)	12(20)	0.07

HFPEF: heart failure with preserved ejection fraction. HFREF: heart failure with reduced ejection fraction.

**Table 2:** Baseline characteristics of heart failure patients

	**Ejection fraction**		
	**HFPEF**	**HFREF**	**p**
Age (median[IQR])	76[67-79]	62[56-69]	0.001
Systolic blood pressure (mmHg) (mean ± SD)	169 ± 45	134 ± 25	0.004
Diastolic blood pressure (mmHg) (mean ± SD)	93 ± 19	88 ± 17	0.31
Heart Rate (b/min) (median[IQR])	88[74-111]	99[80-120]	0.15
NYHA class IV (%)	13(68.4)	45(75)	0.57
Glycemia (mg/dl) (median[IQR])	162[109-243]	132[95-182]	0.09
GFR (mean ± SD)	64 ± 21	65 ± 22	0.79
Galectin-3 (ng/ml) (median[IQR])	9.7[3.7-12]	8.2[3.6-14]	0.96
PWV (m/s) (median[IQR])	11.3[9.4-16]	10[7.3-12.5]	0.04
CD (10^3^ mmHg) (median[IQR])	2.7[1.8-3.5]	3.9[2.7-5.7]	0.009
Young (kPa) (median[IQR])	563[432-749]	408[260-706]	0.15
IMT (cm) (median[IQR])	1.1[0.9-2.2]	1[0.8-1.1]	0.08
E/e' (median[IQR])	10[8-14]	13.6[9.8-16]	0.04
E/Vp (mean±SD)	1.8 ± 0.7	2.4 ± 0.8	0.009
LAvolume (ml/m^2^) (mean ± SD)	76 ± 30	74 ± 26	0.77
PASP (mmHg) (median(IQR))	35[25-46]	30[20-42]	0.4
Ea (mmHg*ml^-1^*m^-2^) (median(IQR))	2.8[2.6-3.9]	2.5[2-3.7]	0.11
Elv (mmHg*ml^-1^*m^-2^) (median(IQR))	2.4[1.9-3.7]	0.9[0.7-1.2]	< 0.001
Ea/Elv ratio (median(IQR))	1.1[1-1.3]	2.5[2-3.3]	< 0.001

HFPEF: heart failure with preserved ejection fraction; HFREF: heart failure with reduced ejection fraction; SD:standard deviation; IQR:interquartile range; GFR: glomerular filtration rate; PWV: pulse wave velocity; CD:carotid distensibility coefficient; IMT: intimal medial thickness; LA: left atrium; PASP: pulmonary artery systolic pressure; Ea: arterial elastance; Elv: left ventricular elastance.

### Baseline characteristics, correlation of vascular indices with echocardiography and clinical parameters

Association between covariates (clinical, echocardiographic) and vascular indices was assessed using the Spearman bivariate correlation. High pulse wave velocity levels were significantly associated with higher EF (r = 0.275, p = 0.01), increased Ea/Elv ratio (r = -0.228, p = 0.04), smaller ventricular diameters (r = -0.247, p = 0.02), and elevated systolic blood pressure (r = 0.236, p = 0.03). Lower carotid distensibility coefficient was associated with higher EF (r = -0.332, p = 0.003), increased Ea/Elv ratio (r = 0.38, p < 0.001), higher age (r = -0.236, p = 0.03), and elevated systolic blood pressure (r = -0.258, p = 0.02) and low septal (e') tissue Doppler velocity (r = -0.235, p = 0.03). Carotid intima-media thickness was significantly correlated with higher age (r = 0.446, p < 0.001) and hyperglycemia (r = 0.268, p = 0.01). Neither of the vascular indices (pulse wave velocity, carotid distensibility coefficient, Young modulus) correlated with serum galectin-3 levels (p = 0.1, p = 0.9, p = 0.6). Regression analysis showed a significant association between elevated galectin-3 levels (> 9.9 ng/ml) and an increased arterial ventricular coupling ratio (Ea/Elv) (p = 0.047, OR = 1.9, 95% CI [1.0-3.6]) (Table 3).

**Table 3:** Regression analysis of the association between elevated galectin-3 levels and markers of ventricular-arterial coupling

	**p**	**OR**	**95% CI for OR**	
			**Lower**	**Upper**
Ea	0.09	0.57	0.30	1.09
Elv	0.07	2.36	0.93	6.00
Ea/Elv	0.047	1.92	1.00	3.67

Ea: arterial elastance.; Elv: left ventricular elastance; OR: odds ratio; CI: confidence interval.

### LV remodeling, arterial remodeling and galectin-3

Assessment of the association between echocardiographic variables and serum galectin-3 levels revealed a strong correlation of the protein with systolic function. More specifically, higher galectin-3 levels were related with lower rates of LV pressure rise in early systole (dp/dt) 524 [262-982] mmHg/s (median and IQR) (p = 0.01 for trend) (versus 682 [340-1882] mmHg/s). When grouped by type of diastolic dysfunction, high levels of galectin-3 were associated with type I diastolic relaxation abnormality defined as E/A < 1 ratio (p = 0.017). A regression model analysis of echocardiographic indices and galectin-3 concentrations revealed that PASP (p = 0.04), E/A ratio (p = 0.001), RWT (p < 0.001), EF (p = 0.02), end-systolic volume (p = 0.01) and end-systolic diameters (p = 0.04) were associated with higher galectin-3 concentrations (Table 4). Increased intima-media thickness was strongly related with concentric LV remodeling which is defined as a RWT index > 0.42 (1.05cm [0.7-1.6], p = 0.016) (Table 5).

**Table 4:** Multivariate regression analysis of galectin-3 levels and echocardiographic markers. Echocardiographic parameters were associated with increased galectin-3 levels (> 9.9 ng/mL)

	**p value**	**OR**	**95% CI for OR**	
			**Lower**	**Upper**
Ejection fraction	0.025	1.23	1.02	1.49
E/e' ratio	0.246	0.85	0.65	1.11
E/Vp	0.371	1.73	0.51	5.79
e'-septal peak velocity	0.144	1.58	0.85	2.92
Early decelaration time	0.391	1.00	0.99	1.01
E/A ratio	0.001	0.12	0.03	0.44
Left atrium indexed volume	0.150	1.02	0.99	1.05
Pulmonary artery systolic pressure*	0.046	1.06	1.00	1.13
Relative wall thickness	0.024	0.00	0.00	0.09
LV end-diastolic diameter	0.073	0.73	0.52	1.02
LV end-systolic diameter*	0.046	1.36	1.00	1.85
LV end-systolic volume	0.011	0.89	0.82	0.97
LV end- diastolic volume*	0.011	1.17	1.03	1.33

OR: odds ratio; CI: confidence interval; E,A: trasmitral velocities; Vp: trasnmitral flow propagation velocity;

* statistically significant.

**Table 5:** Comparison of vascular indices according to echocardiographically determined relative wall thickness index

	**PWV (m/s)**		**CD (10^3^mmHg)**		**Young Modulus (kPa)**		**IMT (cm)**	
	**Relative wall thickness**		**Relative wall thickness**		**Relative wall thickness**		**Relative wall thickness**	
	**> 0.42**	**< 0.42**	**> 0.42**	**< 0.42**	**> 0.42**	**< 0.42**	**> 0.42 **	**< 0.42**
Median	10.2	10.1	3	3.8	520	478	1.05	0.95
p-value		0.52		0.30		0.72		0.02

* PWV: pulse wave velocity; CD: carotid distensibility coefficient; IMT: intimai medial thickness.

### Predictive factors for outcomes

A multivariate Cox regression was carried out to identify independent risk factors for outcomes. High galectin-3 levels (p = 0.038, HR = 3.07, 95% CI 1-8.8) and increased PASP (p = 0.007, HR = 1.06, 95% CI 1-1.1) were found to be independent risk factors for all-cause mortality and readmissions in this model (Table 6).

**Table 6:** Cox multivariable regression of outcome measures in decompensated heart failure patients

	**p value**	**HR**	**95% CI for HR**	
			**Lower**	**Upper**
Age	0.019	1.07	1.01	1.15
NYHA class	0.688	0.85	0.38	1.88
Heart rate at admission	0.535	0.99	0.97	1.01
Glycemia	0.619	0.99	0.99	1.00
Glomerular filtration rate	0.343	0.98	0.95	1.01
Hemoglobyn	0.510	0.90	0.68	1.20
Galectin-3*	0.038	3.07	1.06	8.86
Pulse wave velocity	0.573	0.98	0.91	1.05
Carotid distensibility	0.615	1.09	0.76	1.57
Young modulus	0.433	1.00	0.99	1.00
Ejection fraction	0.826	1.00	0.94	1.07
E/e' ratio	0.590	0.95	0.78	1.14
E/Vp ratio	0.817	0.90	0.37	2.17
e'-septal peak velocity	0.768	1.06	0.71	1.58
Left atrium indexed volume	0.673	1.00	0.98	1.02
Pulmonary artery systolic pressure*	0.007	1.06	1.01	1.10
LV end-diastolic diameter	0.395	0.95	0.86	1.06
LV end-diastolic volume	0.115	1.01	0.99	1.02

* Statistically significant. † LV:left ventricle. ‡ HR:hazard ratio. CI:confidence interval

## Discussion

One major finding of this study was that arterial stiffening is strongly present in the heart failure with preserved EF group. Clinical trials have used different cut-off points for EF (> 40%, > 45%, > 50%) to define heart failure with preserved EF syndrome. In this study we used an EF cut-off point > 40% as in Candesartan in heart failure - assessment of reduction in mortality and morbidity (CHARM) trial.^9^ Pathophysiological aspects of this condition include LV stiffness with reduced compliance (due to hypertrophy, matrix apposition), chronotropic incompetence, pulmonary hypertension and vascular stiffening.^10^ In our study, heart failure with preserved EF patients were older and presented higher blood pressure compared with those with reduced EF, similarly to the data reported in the Acute Decompensated Heart Failure National Registry (ADHERE) database.^11^

Kawaguchi et al.^12^ showed that patients with heart failure with preserved EF have increased arterial stiffening by measuring the arterial elastance. Pulse wave velocity is known to be a marker of arterial stiffening and is widely used in clinical trials. It is of prognostic value and is increased in congestive in heart failure patients.^13^ We have shown that pulse wave velocity is consistently higher in the group with preserved EF than in the group with reduced EF, which is consistent with that reported by Balmain et al.^13^ (10.7 m/s vs.8.9m/s. p < 0.05)^14^. Patients from the group with reduced EF had lower pulse wave velocities probably due to reduced cardiac output and blood pressure. These findings indicate that patients with heart failure with preserved EF have decreased carotid arterial distensibility and advanced age. Kitzman et al.^15^ also reported that carotid arterial distensibility is decreased in older heart failure patients with preserved EF and is correlated with impaired exercise tolerance. An additional finding of this study is that increased carotid intima-media thickness, which is a marker of atherosclerosis, highly correlates with concentric LV remodeling and hypertrophy. Another study, carried out by Xu et al.^16^ on the general Chinese population pointed out that increased carotid intima-media thickness is associated with an increase of the LV mass index and posterior wall thickness.

Galectin-3 has been demonstrated to promote fibrosis via increased activation of the transforming growth factor beta / Smad-3 (TGF-beta/Smad-3) signaling pathway.^17^ Because of its role in fibrogenesis, galectin-3 has been proposed as a potential biomarker involved in both cardiac and vascular remodeling. Increased expression of galectin-3 has been documented in decompensated heart failure patients and was associated with LV remodeling.^18,19^ A recent study carried out by Calvier et al.^20^ has shown that galectin-3 is a mediator of vascular fibrosis. The authors demonstrated that galectin-3 levels are overexpressed by vascular smooth muscle cells in aldosterone treated mice, which in turn increases the deposit of collagen type I in these cells.^20^ Based on these findings and the fact that neurohormonal activation is enhanced in heart failure patients, we tried to establish whether there is a link between serum galectin-3 levels and vascular stiffening indices. Our data showed no correlation between these markers, even though there was a slight increase, but not significant, in arterial rigidity with higher levels of galectin-3. Contrary to these observations, Libhaber et al.^21^ in a community sample study involving 966 participants, showed that galectin-3 was independently associated with carotid-femoral pulse wave velocity.^21^ Our findings are the first to suggest that increased galectin-3 levels are associated with abnormal ventricular-arterial coupling, meaning that it could be involved in the stiffening process of both myocardial and arterial components. Arterial stiffness and reduced cardiac compliance are responsible for increased coupling ratio (Ea/Elv).^22 ^The Ea/Elv ratio is a complex parameter that includes arterial compliance, peripheral vascular resistance, impedance, systolic and diastolic time intervals, LV contractility and LV function.^23^ It can be used to assess the interaction between the myocardium and the arterial system. In heart failure with reduced EF patients, there is an elevation in Ea/Elv ratio due to an increase in the arterial load and a decrease of LV contractility. On the other hand, this ratio is balanced in patients with heart failure with preserved EF compared with normal subjects due to a simultaneous rise in both arterial and LV elastance, which is in line with our findings.^23^

As previously observed by De Boer et al.,^24^ we found that serum levels of galectin-3 did not differ between patients with heart failure with preserved EF and those with reduced EF.^24^ So far, the role of galectin-3 in cardiac stiffness has been shown by its association with echocardiography markers of diastolic dysfunction: high LV filling pressures (expressed by the E/e ratio) and abnormal LV relaxation (e-wave velocity).^25^ As for the systolic function, in a study realized by Van der Velde et al.,^26^ galectin-3 was proven to be an independent predictor for EF in patients after myocardial infarction.^26^ In our study, we observed that high galectin-3 levels were associated with impaired LV systolic function reflected by lower rates of LV pressure rise. To our knowledge, this is the first study to report a correlation of this kind. The rate of LV pressure rise in early systole (dp/dt) assesses global LV contractility by Doppler examination of mitral regurgitation jet. As the contractility force exerted by the LV decreases in advanced heart failure, the rates of LV pressure rise become lower. In addition, since heart failure patients with reduced EF and increased LV diameters have significantly lower dp/dt rates, galectin-3 could be involved in eccentric remodeling, representing a possible marker of severe systolic dysfunction. The negative effects of galectin-3 on systolic function were also seen in increased end-systolic volume and end-systolic diameter. Lok et al.^19^ were the first to demonstrate a positive correlation between high baseline levels of galectin-3 and the increase of end-diastolic and end-systolic diameters over time in heart failure patients. Another interesting finding was that increased arterial pulmonary pressure was predictive of higher galectin-3 serum levels. The association between pulmonary hypertension and galectin-3 has been demonstrated in patients with diastolic heart failure-induced pulmonary hypertension, and galectin-3 levels has been shown to be positively correlated with right ventricular systolic pressure (p<0.01).^27,28^ Pathophysiology of pulmonary hypertension in heart failure is characterized by pulmonary arterial remodeling, in which medial hypertrophy and intimal fibrosis are the main components.^29^ Having this in mind and the fact that diastolic dysfunction is associated with different grades of myocardial hypertrophy and fibrosis, galectin-3 is likely to be involved in myocardial and pulmonary artery remodeling processes. This is of particular importance since it may explain both the active precapillary pulmonary hypertension that plays an important role in the prognosis and worsening of heart failure and the passive postcapillary component as a result of increased end-diastolic pressures.

Here, we report that serum galectin-3 levels above the mean of 10 ng/mL and increased PASP are independent prognostic factors for all-cause mortality and readmissions of heart failure patients over a period of 12 months. Our findings are somewhat similar to those reported in "The N-terminal Pro-BNP investigation of dyspnea in the emergency department (PRIDE)" study^18^ in which the authors noted recurrence of cardiac decompensation and mortality in patients with galectin-3 levels above 9.42 ng/mL. However, the evaluation period was shorter than ours, comprising 60 days. In another study on 592 heart failure patients, baseline galectin-3 levels powerfully predicted outcome over a period of 18 months.^24^ With respect to pulmonary artery systolic pressure, less is known about its prognostic value in left heart failure. De Bursi et al.^30^ were the first to evaluate the impact of PASP on a large community sample of heart failure patients. They indicated that PASP measured by echo-Doppler strongly predicted mortality in these patients, which makes it a relevant indicator of outcome in this population.^30^

## Conclusions

This study was designed to fill the gap in knowledge about vascular remodeling and its progression in heart failure patients, which is considered to be responsible for adverse outcomes. This study showed no significant correlation between serum galectin-3 levels and arterial stiffening markers in this population. Even so, our data suggest that galectin-3 contributes to abnormal ventricular-vascular coupling. Galectin-3 might be involved in vascular and myocardial stiffening of heart failure patients, but larger samples would be useful to confirm the preliminary findings of our study. Also, galectin-3 may be predictive of raised pulmonary pressures and could be responsible for the pathological changes in the distal pulmonary arteries with consequent pulmonary vascular resistance and hypertension described in heart failure. Both galectin-3 and PASP were independent prognostic factors. The present study extends previous findings that arterial stiffening is increased in heart failure with preserved EF and is associated with LV concentric remodeling. Our findings highlight the involvement of galectin-3 in diastolic and systolic impairment, but most importantly, that high galectin-3 concentrations are associated with severe systolic dysfunction.

### Potential limitations

One potential limitation of our study is that the measurement of aortic length to estimate the pulse wave velocity required some approximations due to anatomical changes of the aorta especially in the elderly. As well as that, the estimation of vascular dimensions from the body surface remains a questionable technique. Another limitation is in determining the time taken by the blood ejected from the heart to reach the arteries, given the fact that this parameter is dependent on factors such as conduction abnormalities or rhythm. Since some of our patients presented conduction abnormalities (bundle branch blocks) or atrial fibrillation, several measurements were needed to determine this parameter and to record good pulse waves.

During admission, there might have been some delays from the obtaining of vascular indices and collection of blood samples for galectin-3 determination. This may have affected the assessment of the relationship between these parameters, particularly because the biological half-time of galectin-3 has not been established. However, unlike NT-proBNP which decreases in LV loading, due to its role in fibrosis, galectin-3 does not respond to a hemodynamically compensated condition. For this reason, it would have been more accurate to evaluate whether changes in the concentration of galectin-3 would affect arterial stiffening. In addition, the relatively small size of our cohort limited the evaluation of correlations of galectin-3 with echocardiographic indices. Due to these limitations, we hope that our study serve as a hypothesis-generating basis for further large prospective studies to confirm our findings.
